# From the Trenches: A Cross-Sectional Study Applying the GRADE Tool in Systematic Reviews of Healthcare Interventions

**DOI:** 10.1371/journal.pone.0034697

**Published:** 2012-04-05

**Authors:** Lisa Hartling, Ricardo M. Fernandes, Jennifer Seida, Ben Vandermeer, Donna M. Dryden

**Affiliations:** 1 Department of Pediatrics, Alberta Research Centre for Health Evidence, University of Alberta, Edmonton, Canada; 2 Department of Pediatrics, Cochrane Child Health Field, University of Alberta, Edmonton, Alberta, Canada; 3 Gulbenkian Programme for Advanced Medical Education; Departamento da Criança e da Família (Child and Family Department), Hospital de Santa Maria; Laboratório de Farmacologia Clínica e Terapêutica (Clinical Pharmacology and Therapeutics), Instituto de Medicina Molecular, Lisboa, Portugal; Universidad Peruana Cayetano Heredia, Peru

## Abstract

**Background:**

GRADE was developed to address shortcomings of tools to rate the quality of a body of evidence. While much has been published about GRADE, there are few empirical and systematic evaluations.

**Objective:**

To assess GRADE for systematic reviews (SRs) in terms of inter-rater agreement and identify areas of uncertainty.

**Design:**

Cross-sectional, descriptive study.

**Methods:**

We applied GRADE to three SRs (n = 48, 66, and 75 studies, respectively) with 29 comparisons and 12 outcomes overall. Two reviewers graded evidence independently for outcomes deemed clinically important a priori. Inter-rater reliability was assessed using kappas for four main domains (risk of bias, consistency, directness, and precision) and overall quality of evidence.

**Results:**

For the first review, reliability was: κ = 0.41 for risk of bias; 0.84 consistency; 0.18 precision; and 0.44 overall quality. Kappa could not be calculated for directness as one rater assessed all items as direct; assessors agreed in 41% of cases. For the second review reliability was: 0.37 consistency and 0.19 precision. Kappa could not be assessed for other items; assessors agreed in 33% of cases for risk of bias; 100% directness; and 58% overall quality. For the third review, reliability was: 0.06 risk of bias; 0.79 consistency; 0.21 precision; and 0.18 overall quality. Assessors agreed in 100% of cases for directness. Precision created the most uncertainty due to difficulties in identifying “optimal” information size and “clinical decision threshold”, as well as making assessments when there was no meta-analysis. The risk of bias domain created uncertainty, particularly for nonrandomized studies.

**Conclusions:**

As researchers with varied levels of training and experience use GRADE, there is risk for variability in interpretation and application. This study shows variable agreement across the GRADE domains, reflecting areas where further guidance is required.

## Introduction

The GRADE tool (Grading of Recommendations Assessment, Development and Evaluation) has been developed and refined over recent years by an international working group (www.gradeworkinggroup.org). One of the motivations for developing the tool was to address shortcomings of other approaches to rating the strength or quality of a body of evidence. The GRADE tool offers a “common, sensible, and transparent approach to grading quality of evidence and strength of recommendations”. It represents an important tool for decision-makers as it provides a mechanism to bridge the gap from evidence synthesis to application of the evidence for informed decision-making.

Much has been published about the GRADE tool (www.gradeworkinggroup.org/publications/index.htm). One of the first citations regarding the GRADE tool appeared in the *Canadian Medical Association Journal* in 2003 [1] with a second in the *British Medical Journal* soon after [2]. Recently, a series of publications about the GRADE tool was published in the *Journal of Clinical Epidemiology* [3]–[12]. These reports provide details about the development of the tool and general instructions on how the tool should be applied. GRADEPro software is available which offers a structured format to present the information, with an accompanying handbook that contains instructions and examples (www.ims.cochrane.org/revman/gradepro) [13].

In brief, reviewers assess the body of evidence available for a given question and outcome with respect to several key domains. These include risk of bias, consistency, precision, directness, and publication bias. Additional domains that can be considered are magnitude of effect, confounding, and dose-response relationship. In general, evidence from randomized trials may start at high quality while evidence from observational studies starts at low quality. The evidence can be upgraded or downgraded based on the remaining domains. This results in a final assessment of quality of the evidence into one of four categories: high, moderate, low, or very low. These categories reflect the confidence the assessors have that the evidence represents the true effect and is unlikely to change with future research. Assessors are encouraged to document the reasons for their decisions for transparency.

Use of the GRADE tool has recently been adopted by The Cochrane Collaboration, and grading the evidence is now a recommended step within Cochrane systematic reviews [14]. Summary of Findings tables can be developed using GRADEPro software and integrated into Cochrane reviews. These tables show effect estimates for key outcomes as well as assessments of the quality of evidence based on the GRADE tool. This provides an effective summary of the evidence for the reader. By providing a quality of evidence assessment alongside the effect estimates, the reader has some indication of how much confidence they can place in the findings. Moreover, the reader has a sense of whether “the evidence reflects the true effect” and whether additional research would likely change the effect estimate or the reader's confidence in the estimate [15].

Despite numerous publications about the GRADE tool (including some criticisms [16]), there have been few empirical and systematic evaluations of applying the tool in practice [17]. As grading the evidence is now a recommended step in Cochrane systematic reviews and in other evidence synthesis initiatives outside The Cochrane Collaboration [15], [18], new users with variable levels of training are likely to be applying the tool. The widespread use of the tool by users with variable training has implications for interpretation and application of the tool.

The objectives of this study were to apply GRADE in systematic reviews in order to assess inter-rater reliability and identify areas of uncertainty. Our focus was on use of the tool to rate quality of evidence, not the strength of recommendations. The goal was to identify areas that require further guidance in order to provide clarity for users, particularly systematic reviewers.

## Methods

We applied the GRADE tool to three systematic reviews that we conducted at our research centre [19]–[21]. The systematic reviews were broad in scope and each included several interventions and comparisons. They were on varied clinical topics and involved different types of interventions (e.g., pharmacological and non-pharmacological) and outcomes. Further, one included only randomized controlled trials (RCTs) while the other two also included select nonrandomized study (NRS) designs. The topics of the systematic reviews were: 1) steroids and bronchodilators for bronchiolitis; 2) operative and nonoperative interventions for rotator cuff tears; and, 3) pain management interventions for hip fracture (Table 1).

**Table 1:** Comparisons and outcomes examined in the systematic reviews.

Review	Comparisons	Outcomes
Case 1: Steroids and bronchodilators for bronchiolitis [19]	• steroid vs. placebo	• admissions
	• epinephrine vs. placebo	• length of stay
	• salbutamol vs. placebo	• clinical score
	• epinephrine vs. salbutamol	• adverse events
	• epinephrine and dexamethasone combined vs. placebo	
	• epinephrine and dexamethasone combined vs. salbutamol	
Case 2: Operative and nonoperative interventions for rotator cuff tears [20]	• early vs. late surgical RCR	• health-related quality of life
	• open vs. mini-open RCR	• function
	• mini-open vs. arthroscopic RCR	• time to return to work
	• open RCR vs. arthroscopic RCR	• cuff integrity
	• open or mini-open RCR vs. arthroscopic RCR	
	• open RCR vs. open or arthroscopic debridement	
	•	
	•	
	• arthroscopic RCR vs. RCR plus acromioplasty	
	• arthroscopic RCR vs. acromioplasty alone	
	• single-row vs. double-row suture anchor repairs	
	• mattress vs. simple stitch	
	• continuous passive motion with physical therapy vs. physical therapy alone	
Case 3: Pain management interventions for hip fracture [21]	• analgesia vs. other	• acute pain
	• spinal vs. general anesthesia	• chronic pain
	• continuous vs. single administration of spinal anesthesia	• mortality
	• addition of fentanyl to spinal anesthesia	• incidence of serious adverse events
	• addition of morphine to spinal anesthesia	
	• addition of sulfentanil to spinal anesthesia	
	• comparative alternative medicine	
	• nerve blocks vs. no block	
	• nerve blocks vs. regional anesthesia	
	• neurostimulation	
	• rehabilitation	
	• traction	

RCR = rotator cuff repair.

For each review, two reviewers independently graded the evidence for key outcomes. The outcomes were selected a priori by the team of investigators associated with each review based on clinical importance. In general the team of investigators for each review included at least two clinicians with expertise in the area and at least one methodologist with expertise in research methods and systematic reviews. Assessments were made for the four main domains (risk of bias, consistency, directness, and precision), as well as overall quality of evidence (high, moderate, low, very low [or insufficient]) (Table 2). The GRADE tool includes a fifth main domain (publication bias); however, we followed guidance set out by the Agency for Healthcare Research and Quality Evidence-based Practice Center Program which considers publication bias an optional domain [15]. Further, the GRADE tool has additional domains, including dose-response association, possible confounding, and strength of association (magnitude of effect); we chose to focus on the main domains for this study.

**Table 2:** GRADE domains and assessment options*.

Risk of bias	Consistency	Directness	Precision	Overall Strength of Evidence
• High	• Consistent	• Direct	• Precise	• High
• Medium	• Inconsistent	• Indirect	• Imprecise	• Moderate
• Low	• Unknown or not applicable			• Low
				• Insufficient (very low)

* Based on Owens et al. [15].

Four individuals were involved in assessing the quality of evidence. All individuals had substantial experience and/or training in systematic review methods. LH has doctoral level training in research methods, more than 10 years of SR experience, and clinical training and experience in rehabilitation medicine. DD has doctoral level training in epidemiology and more than 5 years of SR experience. RF is a practicing pediatrician currently completing a doctoral degree with 4 years of SR experience. AA-S is a physician with specialty training, a doctoral degree in research methods, and 6 years of SR experience. The reviews were assessed by LH and RF (bronchiolitis), LH and DD (rotator cuff), and DD and AA-S (hip fracture). All reviewers read and were familiar with guidance on applying the GRADE tool [13], [15].

Within reviews, we calculated inter-rater reliability between reviewers for each domain and overall quality of evidence using kappa statistics. Agreement was categorized as poor (0.00), slight (0.01–0.20), fair (0.21–0.40), moderate (0.41–0.60), substantial (0.61–0.80), or almost perfect (0.81–1.00) using one of the standard approaches [22]. We were unable to calculate kappa if one or both reviewers in the pair always gave the same assessment for the domain (e.g., directness always assessed as direct). In this case, we calculated the percent of assessments within the domain for which the two reviewers agreed. For each domain, we identified items that may have led to discrepancies between reviewers. We have presented this information in a narrative summary.

## Results

Overall results are presented in Table 3 and are described by review and by domain below.

**Table 3:** Inter-rater reliability and agreement for GRADE domains and overall strength of evidence.

Domain	Case 1 [19]		Case 2 [20]		Case 3 [21]	
	Kappa or percent agreement	Level of agreement based on kappa	Kappa or percent agreement	Level of agreement based on kappa	Kappa or percent agreement	Level of agreement based on kappa
Risk of bias	0.41	Moderate	33%	n/a	0.06	Slight
Consistency	0.84	Almost perfect	0.37	Fair	0.79	Substantial
Directness	41%	n/a	100%	n/a	100%	n/a
Precision	0.18	Slight	0.19	Slight	0.21	Fair
Overall strength of evidence	0.44	Moderate	58%	n/a	0.18	Slight

### Case 1: Steroids and bronchodilators for bronchiolitis [19]

We included 48 RCTs in this review. We graded the evidence for six comparisons and four outcomes (Table 1). Further, we pooled and graded the evidence separately for inpatient and outpatient populations. There were a total of 51 assessments. Inter-rater reliability ranged from slight for precision (κ = 0.18) to almost perfect for consistency (κ = 0.84). Reliability for overall quality of evidence was moderate (κ = 0.44).

### Case 2: Operative and nonoperative interventions for rotator cuff tears [20]

This review included 27 trials (21 RCTs and 6 controlled clinical trials) and 39 cohort studies. We graded evidence for 11 comparisons and four outcomes (Table 1). There were a total of 24 assessments. Inter-rater reliability was calculated for two domains and was slight for precision (κ = 0.19) and fair for consistency (κ = 0.37). Reliability could not be assessed for the other domains or overall strength of evidence, as one rater assessed all the same. Agreement for these domains was 33% for risk of bias, 100% for directness, and 58% for overall quality of evidence.

### Case 3: Pain management interventions for hip fracture [21]

This review included 65 RCTs and 10 cohort studies. We graded evidence for 12 comparisons and four outcomes (Table 1) for a total of 36 assessments. Inter-rater reliability ranged from slight (κ = 0.06) for risk of bias to substantial (κ = 0.79) for consistency. Reliability for overall quality of evidence was slight (κ = 0.18). There was 100% agreement between reviewers for directness.

### Sources of disagreement by domain

#### Risk of bias

One of the key challenges that arose during assessment of this domain was how to integrate risk of bias or quality assessments of the individual studies. This was more straightforward for RCTs than NRS. For instance, if the majority of evidence came from RCTs assessed as low risk of bias based on the Cochrane Risk of Bias tool [14], then we assessed the overall body of evidence for the comparison and outcome of interest as low risk of bias. That is, there was room for a fairly direct translation from assessments using the Risk of Bias tool to the risk of bias domain. However, there was more discrepancy when other quality assessment tools were used, particularly for NRS. For example, questions arose regarding how to translate a range of scores for cohort studies on the Newcastle Ottawa Scale [23] (from 0 to 9) into one of three risk of bias classifications. Moreover, the move away from overall quality scores or summaries towards a component approach for individual studies [14] was found to be incongruous with forcing an overall assessment of the risk of bias for a group of studies.

Another question that arose was whether there should be pre-specified starting points for different study designs. For instance, it was questioned whether RCTs should always start out as low risk of bias overall whereas all NRS start out as high risk of bias overall. One restriction that we noted within the GRADE recommendations is that NRS are considered as a homogeneous body and start at high risk of bias. Moreover, they are all referred to as “observational studies.” This does not distinguish between studies that may be more or less prone to bias based on design features, such as prospective comparative studies versus a single sample before-after study. We questioned whether there should be a distinction among “observational studies” where some would start at high risk of bias because of key design limitations whereas others would start at moderate risk of bias based on more rigorous methodological approaches. For example, in the review of operative and nonoperative interventions for rotator cuff injuries, we considered prospective cohort studies that controlled for confounding to be less prone to bias and graded these higher than retrospective studies that failed to control for key confounders.

A final key question that arose for the risk of bias domain was whether there should be different thresholds for different study designs. For instance, should RCTs always be higher in terms of risk of bias compared with NRS. Further, should the minimum threshold for RCTs be moderate risk of bias because the use of an appropriate randomized design always ensures less risk of bias compared with NRS. Alternatively, is it ever appropriate to rate NRS as low risk of bias given that they do not employ randomization and are always prone to bias due to potential imbalance between groups being compared, even when rigorous design or analytic methods are used to attempt to control for confounding.

#### Consistency

When a meta-analysis was available for the comparison of interest, we relied heavily on the I^2^ statistic, which provides an indication of the extent of statistical heterogeneity across a set of studies [14]. As a general guideline if I^2^ was greater than 50%, the results were considered inconsistent. Similarly if I^2^ was less than 20%, the results were considered consistent. However, these were general guides and were not consistently applied; there were also areas where judgment was required. More disagreement arose when there was no meta-analysis. In general, the reviewers took a qualitative approach to assessing consistency across studies. For example, they considered whether the estimates were similar in terms of magnitude and direction of effect, as well as statistical significance. The somewhat different approach with and without meta-analysis raises the issue of whether assessments are made with differential standards depending on whether a meta-analysis is done. Often the reason for not conducting a meta-analysis, or pooling data, is due to heterogeneity across studies; hence, one option is that the default is “inconsistent” or “unknown” when there is no meta-analysis.

#### Directness

The two main criteria we used to assess this domain were whether the outcomes were intermediate or surrogate rather than the final health outcome, and whether the evidence came from direct head-to-head comparisons. We found that determining whether an outcome was intermediate or surrogate was somewhat context-specific and dependent on the research question posed in the review. For example, pain may be considered a surrogate or an intermediate outcome related to function. In the hip fracture review, pain itself was a primary outcome of interest, whereas in the rotator cuff review, pain was intermediate and function was among the primary outcomes of interest. We felt that directness with respect to whether the outcome is intermediate or surrogate could be determined at the outset of the review, thus eliminating disagreement at the stage of grading the evidence.

We did not assess for applicability within this domain as there are discrepancies in current published guidance for grading evidence. One approach restricts the assessment to the two main criteria above [15], whereas the other approach includes considerations of generalizability or applicability of the findings [13]. The first approach considers generalizability outside the context of grading the evidence and within the context of making recommendations for application of the evidence, whereas the second would consider an indirect population as a possible source of indirectness.

#### Precision

This domain consistently had low reliability. Assessments were particularly problematic when no meta-analysis had been conducted as there was no confidence interval around a single estimate to determine the inclusion or exclusion of no effect or important benefit/harm. Similarly, assessment of precision was difficult when multiple meta-analyses were available for a given outcome due to differences in how an outcome was measured across studies. For instance, precision may have varied between a meta-analysis of pain measured on a continuous scale and a meta-analysis reporting the proportion of patients experiencing pain. In these cases, we relied heavily on the minimum thresholds for number of events (dichotomous outcomes) or subjects (continuous outcomes) that are specified in the GRADEPro Handbook [13].

We found it critical to set, a priori, a clinical decision threshold [12] for each outcome. This is not currently a standard step within systematic reviews. Furthermore, in some clinical areas there is not a well-defined or generally accepted threshold; in other cases these are based on expert opinion or consensus statements and have not been validated in patient-oriented outcomes. There is a need to develop this information for different clinical areas and guidance is needed for how this should be done.

**Figure 1:**
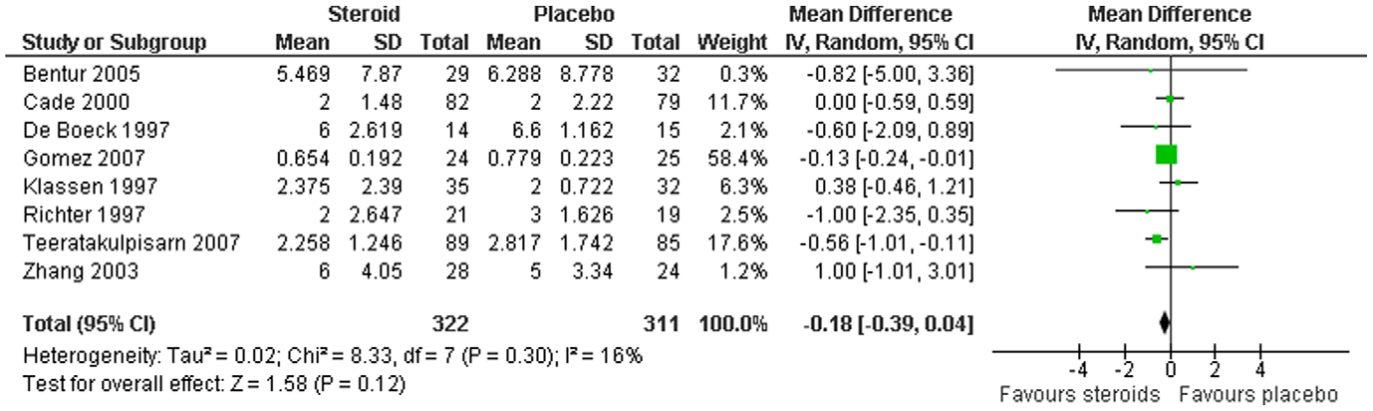
Example of meta-graph showing no difference in length of stay between treatments.

Much of the guidance in the GRADEPro Handbook is directed toward assessing precision around evidence of effect [13]. There was, however, a lack of clarity around whether the same rules apply regarding evidence of no effect. Figure 1 illustrates a finding of no difference between treatment and placebo with a narrow confidence interval that does not include an important difference. Further, the total sample size exceeded the stipulated threshold of 400. However, we were unclear whether the same thresholds applied in order to rate the finding as precise or whether different sample size thresholds were needed akin to differences in sample sizes for trials with different hypotheses (e.g., superiority, inferiority, non-inferiority, equivalence).

#### Overall quality of evidence

We found inconsistencies in our approach to upgrading and downgrading as well as the extent of upgrading and downgrading. For the most part, we used study design as our starting point and downgraded by one step for each domain that was not met; this approach appears to be consistent with the GRADE recommendation [13]. There were discrepancies between reviewers in the bottom threshold of overall quality: some reviewers felt that if there was some evidence (even from one study), the overall quality should be rated “low”, while “very low” (alternatively referred to as “insufficient” [15]) should be reserved for cases where there was no evidence at all.

### Other general observations

As we gained experience with applying GRADE for various review topics which had different questions and different types of evidence, we questioned whether we should aim for consistency across different bodies of evidence. For instance, should the evidence for the rotator cuff project, which was generally based on weak study designs with high risk of bias, ever be rated the same or higher than topics where the evidence comes only from RCTs. For instance, in the bronchiolitis review, some of the evidence was graded as low because it was based on a single trial, therefore the consistency was unknown and the confidence interval just crossed the null. However, the trial was at low risk of bias and was adequately powered to detect differences in the outcome of interest. Moreover, it was the largest trial conducted to date in the field, in some cases exceeding the sample size of all the other trials combined. Yet, the grade of evidence was comparable or in some cases lower than the body of evidence for the rotator cuff review which was driven by NRS.

Two additional points were raised during the course of our work. First, we questioned whether we should develop strict rules for application of the GRADE tool in the work at our centre with the intent of increasing agreement between reviewers and across reviews; or, alternatively provide general guidance and reach consensus within the different reviews or clinical/topic areas. The latter approach may yield more inconsistency across reviews although this may provide important information for the end-user. For instance, if the reviewers report their agreement, the reader can judge the extent to which the specific issue is open to judgment and interpret the evidence more or less cautiously based on this information.

The second additional point was challenges with terminology. We found it confusing for the risk of bias domain to use the same terminology as the process of assessing risk of bias of individual studies. Similarly, the overall quality of evidence was at times confused with quality of studies. Moreover, guidance documents differentially referred to the same overall assessment as quality of evidence [13] and strength of evidence [15].

## Discussion

Rating a body of evidence using the GRADE tool is becoming an important and recommended step in systematic reviews and other evidence synthesis initiatives. Rating the quality of a body of evidence is valuable for the end-users of evidence syntheses as it provides an indication of the confidence they can place in the results. Moreover, it is a key step in translating the body of evidence into clinical practice guidelines. We undertook this study to identify areas of uncertainty in applying the GRADE tool in order to inform the evidence synthesis efforts at our centre. Our research identified key areas where further guidance is needed to inform the application of the tool. Specifically, the domains of precision and risk of bias created the most inconsistency and challenges, whereas directness and consistency were more straightforward (although still had substantial disagreement at times). Further, we encountered challenges when there were no meta-analyses upon which to base assessments, when outcomes were measured in different ways, and when evidence included nonrandomized studies.

Application of the GRADE tool is complex as evidenced by the series of 20 articles that are currently being published in the *Journal of Clinical Epidemiology* [11]. It is clear that both methodological and clinical expertise is required when applying the GRADE tool. Further, we believe the GRADE developers would agree that specific training in use of the GRADE tool is recommended. Our concern is that with widespread adoption of the GRADE tool in Cochrane systematic reviews and non-Cochrane synthesis efforts, there will be inconsistent application of the tool and context-specific decision rules that emerge. Moreover, different organizations or researchers may adapt the GRADE approach for their specific interests, as has already occurred 15,24. This has the potential to yield discordant assessments, which will ultimately affect those using this information to make decisions and recommendations for clinical care. To alleviate some of these concerns, all reviews using tools such as GRADE should have a minimum of two reviewers independently apply the tool, their agreement should be reported, and the basis for their decisions should be transparent.

### Strengths and limitations

This is one of the first systematic and empirical evaluations of applying the GRADE tool in systematic reviews. We recognize that the tool is not meant to eliminate disagreements nor the need for judgments [11]. Our intent in measuring agreement was to identify areas of greatest uncertainty and explore reasons for the discrepancies. We graded the evidence for three systematic reviews; while the number of reviews was small, there were numerous comparisons and outcomes within each review (29 comparisons and 12 outcomes overall). The reviews also represented a variety of clinical topics and types of evidence. The reviewers who applied the GRADE tool had not undergone formal training in the use of the tool; however, the reviewers all had extensive experience and training in research methods and specifically systematic reviews. We expect that many individuals who apply the GRADE tool may also do so without formal training. Hence, our experience reflects what we are likely to see in practice. Finally, we did not assess the domain of publication bias. This is specified as one of the main domains in the GRADEPro Handbook but not by other guidance documents, and specifically the guidance offered by the stakeholder group for two of our three reviews [15]. Nevertheless, in most cases, we did not have sufficient numbers of studies within outcomes and comparisons to formally test for publication bias.

### Conclusions

As the GRADE developers acknowledge, “refinements are inevitable” for new and innovative tools such as GRADE [11]. The intent of our research was to describe our experience applying to GRADE tool and to document the areas that created uncertainty and where clarity and guidance are needed. We trust that our results will serve to optimize the utility of GRADE as a tool for evidence synthesis and practice recommendations.
